# Mining chemical information from open patents

**DOI:** 10.1186/1758-2946-3-40

**Published:** 2011-10-14

**Authors:** David M Jessop, Sam E Adams, Peter Murray-Rust

**Affiliations:** 1 Unilever Centre for Molecular Science Informatics, Department of Chemistry, Lensfield Road, Cambridge CB2 1EW, UK

## Abstract

Linked Open Data presents an opportunity to vastly improve the quality of science in all fields by increasing the availability and usability of the data upon which it is based. In the chemical field, there is a huge amount of information available in the published literature, the vast majority of which is not available in machine-understandable formats. PatentEye, a prototype system for the extraction and semantification of chemical reactions from the patent literature has been implemented and is discussed. A total of 4444 reactions were extracted from 667 patent documents that comprised 10 weeks' worth of publications from the European Patent Office (EPO), with a precision of 78% and recall of 64% with regards to determining the identity and amount of reactants employed and an accuracy of 92% with regards to product identification. NMR spectra reported as product characterisation data are additionally captured.

## Background

The enormous increase in the output of scientific data in recent times now requires radical changes in the way in which it is handled. The CAplus database [1] holds more than 32 million references to patents and journal articles and indexes more than 1500 current journals on a weekly basis, while the CAS REGISTRY [2] holds more than 54 million chemical compounds and the CASREACT [3] database more than 39 million single and multi-step reactions. Such resources are created by a labour-intensive process of manual curation with the consequence that a researcher must pay to access them, and the data themselves become a valuable commercial entity. By necessity, this is *closed data*.

The availability of data is vital for data-driven science such as spectra prediction and Quantitative Structure-Activity Relationship (QSAR) modelling, which has become increasingly important to the pharmaceutical industry as it seeks to control the spiralling costs of drug development. *Open data *- data that is freely available to the community-supports and enables such work. The more the culture of Open data spreads, the more such work becomes viable.

This use of Open data for research, though powerful, is not the end of the story. Tim Berners-Lee first described the concept of the Semantic Web [4]. The idea is simple-the World Wide Web comprises a vast collection of information, but information that is largely meaningless to a computer. If it were to be made machine-understandable, then software agents could be developed that would be able use this information as a basis for reasoning and to make decisions. This concept, tied to that of Open data, would allow for computerised scientists conducting their own data-driven research and reporting their conclusions back to humans. The concept of a machine performing research is not one for the world of science fiction-indeed, the robot scientist Adam has conducted its own hypothesis-driven research, reaching conclusions that were later validated by human researchers [5].

In order to make our information machine-understandable, it is necessary to formalise the semantics of the medium in which it is stored. For the semantic web, such formalisation is typically performed by encoding the data using eXtensible Markup Language (XML). The *de facto *standard XML dialect for chemistry is Chemical Markup Language (CML) [6-10]. By rendering chemical information machine-understandable, CML allows for the creation of systems that integrate data of a variety of types and from a variety of sources to perform novel research-a semantic web for chemistry. Datuments [11,12], hyperdocuments for transmitting 'complete' information including content and behavior, can record and reproduce experiments and act as a lossless way of publishing science. Conventional publication paths discourage the full publication of the scientific record-the process itself militates against datuments. Although there is no technical reason for the separation of 'full-text' and 'supporting information', the author is required to recast their information into models that conform to the publisher's technology and business model. A common feature of all mainstream science publication is the universal destruction of high-quality information. Spectra, graphs, *etc*., are semantically rich but are either never published or must be reduced to an emasculated chunk of linear text to fit the paper model. But now we have the technology to address this. Machine-understandability requires both ontological (meaning) and semantic (behaviour) support, and XML is now mature enough that this is possible. Many information components in a datument can be recast as context-free XML and integrated with XML text and XML graphics. Some publishers are actively embracing enhancements to journal articles (see *e.g*. the RSC's Project Prospect [13-15]), and the Chemistry Add-in for Microsoft Word (sometimes referred to as Chem4Word) [16] supports the authoring of chemical datuments using one of the world's most popular word processing packages. The evolution of '(hyper)activated' journal articles is discussed in a further article in this issue [17].

In the absence of author or publisher-led markup, one way in which semantic data collections can be created is through the application of text mining software to the available literature. Chemistry-specific text mining software has been under continuous development at the Unilever Centre over the past decade. A suite of tools have been developed and released, including the named entity recognition tool OSCAR [18-20], the syntactic analysis tool ChemicalTagger [21,22] and the name-to-structure conversion tool OPSIN [23,24]. The availability of these mature, Open packages allow for the large-scale extraction of chemical data from the published literature.

During this work, we have particularly concentrated on chemical texts which share a common style and vocabulary. The most frequently published chemical "chunks" occur in records of chemical synthesis in journal articles, lab books, theses, reports and chemical patents. Of these, legal and contractual restrictions forbid our text-mining of most scientific articles, while lab books and theses are disorganised and difficult to find, even in institutional repositories. We have therefore developed our chemical reaction text mining on the corpus of public patent data. It is a built-in feature of the patent process that the contents of a patent must be published and Openly available after the appropriate period, so in this sense it is an excellent corpus.

A project at the Chemical Abstracts Service (CAS) in the 1980s aimed to produce a system capable of automating or partially automating the indexing process by application of Natural Language Processing (NLP) technologies [25-27]. This system was claimed to "satisfactorily" process 36 out of 40 synthetic paragraphs from the Journal of Organic Chemistry [24] and to produce "usable results" for 80-90% of simple synthesis paragraphs and 60-70% of complex paragraphs [26], where complex paragraphs are defined as describing general procedures, instances of general procedures, analogous syntheses and parallel syntheses. The size of the corpus used to produce this second set of results was not given, nor in either case was the procedure used for corpus creation. Accordingly, it is not possible to regard this area as a solved problem.

The era in which the aforementioned technology was developed was very different. As a division of the American Chemical Society, CAS was in the privileged position of having access to a large body of published work in an electronic format. The situation today is different-the ubiquity of electronic publication and explosion of the scale of publication has granted such access far more widely, though publishers may very well supply the works subject to restrictive terms of use. The chemical patents used in the current work, however, are subject to no such restrictions and so the time for a re-examination of the subject of automated extraction of chemical reactions has come.

The automated extraction of reaction information from the literature will prove highly useful to, for example, the EPSRC's "Dial-a-Molecule" grand challenge, which aims to make the synthesis of a novel compound a quick and efficient process that can be completed in days, not years. The automated prediction of synthetic pathways will require an appropriate reaction database which is not currently available. We estimate that around 10 million syntheses per year are currently published in the literature, and so text-mining is an obvious means by which such information can be obtained.

The approach applied here is similar to that of CrystalEye [28], where we have built tools that retrieve and extract crystallographic data from public sources. This activity has now generated about 250,000 datasets and runs essentially automatically every night. There is no technical reason why an Open patent service should not run in the same way, downloading the incremental updates on the sites at appropriate intervals according to the publishing schedule of the patent organisation in question. The main difference between these activities is that the crystallographic data is already in quasi-semantic form (*i.e*. CIF) and the process is completely algorithmic. With patents there is a variability due to the different styles of natural language and approaches to document layout taken by applicants, and the different technologies used to create the patent itself. However, in practice, most chemical patents have a very closely-defined structure and style of presentation.

## Current systems for automatic analysis of patents

Referees have asked us to comment on the current approaches of Chemical Abstracts and other commercial organizations. Since this is a competitive area it is likely that precise methodology is a trade secret. When one of us (PM-R) heard CAS present at ACS in 2009, the presentation showed that patent analysis was through experts annotating patents with handwriting. PM-R asked about machine methods and was not given a public indication that they were significant. The major semi-public organization is the Fraunhofer Institute which develops its own in-house methodology (which includes OPSIN and OSCAR3 [29]) and publishes accounts of some of its work.

The motivation of the current work is to explore the semantic structure of patents and parts of the semantic chemical information. We have deliberately not used information which would require OCR of text and to this extent we are likely to show an improvement over the documents published in the last century. We have also not addressed Markush structures as there are other publications describing these and some early commercial applications [30,31]. The primary emphasis has been to show that chemical reactions can be turned into semantic form with an acceptable success rate. This article outlines the steps and likely success of others wishing to enter this area.

## PatentEye

The liberation of scientific data and its conversion to machine-understandable forms holds great promise. A key part of the chemical sciences are the reactions that chemists perform and report in great number, and the goal of PatentEye is to demonstrate the potential to create an automated system capable of extracting reactions from the literature, creating machine-understandable representations using CML and sharing them as Open Data. This system is presented as a proof-of-concept, not as a sustainable resource. To increase the reliability of the extracted syntheses, PatentEye attempts to validate the identified product molecules. This validation is achieved by comparison of a candidate product molecule with any accompanying structure diagram using the package OSRA [32-35] for image interpretation and with any accompanying NMR and mass spectra, using the OSCAR3 data recognition functionality. The identified NMR spectra are considered to be valuable data in their own right and are extracted and retained for use in later works.

### Patent documents

Patents are made available on the World Wide Web by a number of patent offices. For legal reasons, they are frequently published as image-based facsimile reproduction of the original document, and are commonly also available as recovered, free-text documents. While the World Intellectual Property Organisation (WIPO) publishes such documents in HTML with minimal markup indicating the position of document sections and headings, both the European Patent Office (EPO) and United States Patent and Trademark Office (USPTO) employ XML formats in which major sections and heading titles are explicitly delimited. The XML formats used by the USPTO and EPO are similar though not identical, and reflect the structure of a patent as agreed by the Common Application Format (CAF) [36]. While only EPO documents were used in the current work, much of the methodology employed is applicable to alternative document sources. In particular, USPTO documents are available for bulk download via Google patents [37] and present an attractive target for text mining. At the time of writing the documents available for download date from 1976 to the present day, and are claimed to number approximately 7 million, across all subjects.

**Figure 1:**
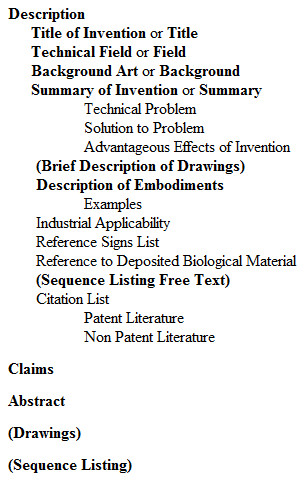
**The standardised patent heading titles, as mandated by the Common Application Format**.

CAF, agreed in 2007 by the EPO, USPTO and Japan Patent Office (JPO), is intended to "simplify and streamline application filing requirements in each Office to allow applicants to prepare a single application in the common application format for acceptance in each of the three Offices" [32]. It mandates the section titles, and their ordering, that are to be used in patent applications. These are shown in Figure 1, in which those titles shown in bold indicate titles that must be included, and those shown in both bold and parentheses must be included where corresponding information is present.

#### Anatomy of a patent and tractability for linguistic tools

Patents are generally large documents, often running to several hundred pages in length. For that reason automated analysis tools are potentially extremely valuable in rapidly exploring their content. Chemical patents are remarkable in that they not only form a large subject domain within the patent literature, but also in that certain sections exhibit a high degree of similarity across the field, particularly for those that discuss the synthesis and properties of organic molecules. This homogeneity makes them very tractable to linguistic analysis.

**Figure 2:**
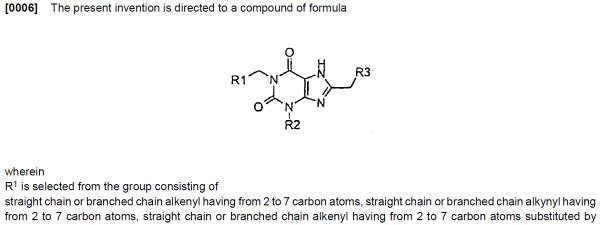
**Typical usage of Markush structures in chemical patents **[45]. Definitions of pseudoatoms (*e.g*. "R1" are frequently several pages in length and are commonly iterative.

The 'Summary of Invention' section is often very long and formulaic. In chemical patents, the subjects of the invention are generally presented in the form of Markush structures, generic chemical structures typically defined by a specific scaffold bearing a number of variable substituent groups, such as that shown in Figure 2. At present, this is too complex for analysis, except for localised sections where OSCAR and OPSIN can recognise catalogues of substituent groups.

**Figure 3:**
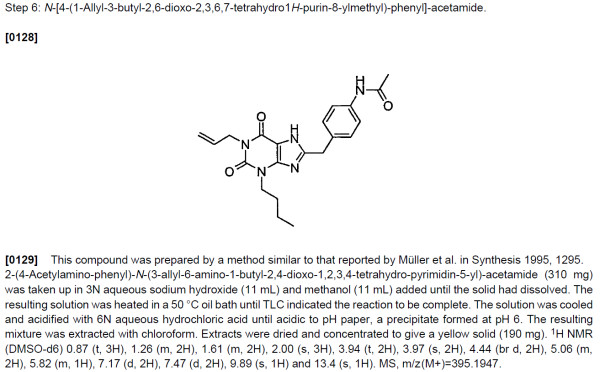
**Typical synthesis report **[34]. The numbers in square brackets indicate sequential numbering of each of the paragraphs in the document.

The examples of the invention that are required to be presented in the description section typically consist of reports of the synthesis of specific compounds that correspond to one of these Markush structures. Such reports appear very much as they would in other parts of the chemical literature such as journal articles and theses, and sometimes, though not always, are accompanied by chemical structure diagrams or characterisation data. An example of such a report is shown in Figure 3. For many patents, these reports can be automatically interpreted with a high degree of precision and recall, and this task represents the major body of work reported in this paper.

Many reactions are described as small variants on a common theme, and so full detail is omitted from the patent document. Typical formulations used for this purpose include "following the procedure for..." or "prepared as in example X...". The challenges to automatically interpreting such examples are to identify the archetypal reaction from the linguistic form and to determine which components of the reaction have been changed in the synthesis. We have made significant progress in interpreter and resolver for this type of language, and in a limited number of cases we have shown that it is possible to not only follow the back-references but replace the chemical structures in context. The process involves a large number of steps and the technology is currently insufficiently mature to be considered a production system.

It is worth noting that identifying sections of the document is not trivial because different applicants use different terminology and often do not announce major sections with the accepted phraseology. Therefore we rely heavily on linguistic processing to determine where sections in the patent begin and end. The patent is also relatively 'flat' in that the humans marking up the patent are only required to identify paragraphs and not subject sections, though some of the high-level document structure illustrated in is explicitly defined in the EPO's XML patent documents. The content of these files is governed by a Document Type Definition (DTD) file that can be downloaded from the EPO website [38]. The root element of the XML documents is ep-patent-document. The common children of this element include SDOBI, abstract, description, claims, ep-reference-list. The only required child of ep-patent-document is abstract, although the other children mentioned will generally be present as well-description, for example, will in practice only be absent in those documents that do not contain a description of the invention *e.g*. patent search reports.

The abstract element can be composed either of an abst-problem and an abst-solution element, or of one or more p elements. The abst-problem and abst-solution elements themselves consist of one or more p elements. The p (paragraph) elements contain text as well as formatting tags such as br and sup that perform the same roles as their namesakes in HTML, and further elements such as tables, maths and chemistry that enclose further content of a specific type.

The Sub-DOcument for BIbliographic (SDOBI) data uses proprietary tags to encode a wealth of metadata related to the patent, *e.g*. the tag B110 contains the patent number, B140 contains the date of publication of the patent and B542 contains the title of the patent. The specification of these tags is contained in the patent DTD, and is beyond the scope of the current discussion.

By convention, each document contains three claims sections-one each in English, French and German. Each claims element contains one or more claim elements, and each claim element contains one or more claim-text elements. A claim-text element is composed of text, HTML-style formatting tags and further tags in a similar manner as for the p element.

The ep-reference-list element contains one or more sets of a heading element followed by one or more p elements. The heading elements contain text and HTML-style formatting tags, and the p elements have been discussed previously.

The description element contains the majority of the text of the patent, and the DTD allows it to be composed of one or more sets of a heading followed by a number of p elements. The DTD also allows for a number of elements to be used that correspond to well-defined sections of the patent document, *e.g*. technical-field, industrial-applicability and description-of-embodiments-unfortunately the comments in the DTD state that "these elements must NOT be used by contractors" and they do not occur in the patents that comprise the corpus used in the current work. As a result, the identification of the different sections of the patent documents is not the trivial task that the DTD allows for, and their boundaries must instead be inferred from the document content.

The XML patent documents are available for download from the EPO website as part of a ZIP package that also contains the PDF facsimile representation and individual TIFF image files that contain the individual figures from the document. The names of these files are numbered sequentially to give identifiers that are referenced in the XML patent document, to indicate which figure occurs at which position in the document.

### PatentEye workflow

**Figure 4:**
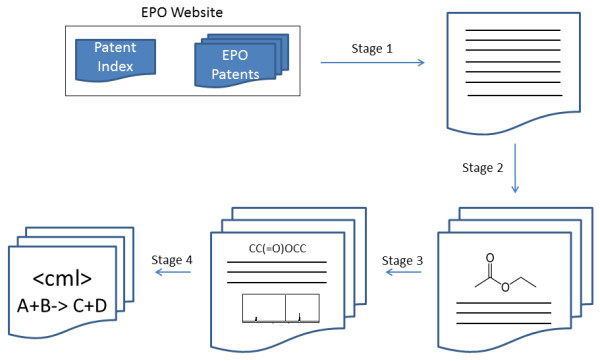
**Schematic workflow for extraction and interpretation of chemical reactions in patents**. Stage 1 -the patent is identified and downloaded. Stage 2-the document is deflattened and segmented. Stage 3-various tools (OPSIN, OSRA, OSCAR3) are used to identify key elements in the reaction and convert them to semantic form. Stage 4-ChemicalTagger is applied to the language of the chemical reaction to determine the roles and processes. Where successful, the extracted information is converted to reactions expressed in CML.

The implemented system is automated to the degree that it is capable of operating with minimal user interaction, and consequently the PatentEye workflow consists of a number of stages of processing. First, chemical patents are identified within the online archive of the European Patent Office (EPO) and are downloaded. The XML documents supplied by the EPO are then semantically enhanced so as to delimit sections and subsections of the text and to introduce additional metadata such as SMILES strings representing the content of structure diagrams and OSCAR3 data markup to describe identified spectra. Finally, reactions are extracted from these semantically enhanced documents using ChemicalTagger and are converted to CML. The overall workflow is depicted in Figure 4.

### Automated identification and download of patents

The European Patent Office (EPO) publishes patent documents through the European Publication Server, hosted at https://data.epo.org/publication-server/. An interactive search using various parameters (a patent ID, a date range within which to search and a list of document kinds) may be performed; alternatively, a weekly digest of patent index files is also provided https://data.epo.org/publication-server/data-coverage. These summaries include the International Patent Classification (IPC) codes assigned to each document. The IPC is a subject-based, hierarchical classification scheme describing the topics covered in a patent. This allows automatic identification of documents relevant to the current work, as listed in Table 1.

**Table 1:** Relevant IPC codes

IPC Code	Description
C07B	General methods of organic chemistry; apparatus therefor
C07C	Acyclic or carbocyclic compounds
C07D	Heterocyclic compounds
C07F	Acyclic, carbocyclic or heterocyclic compounds containing elements other than carbon, hydrogen, halogen, oxygen, nitrogen, sulfur, selenium or tellurium

Once a list of relevant documents has been determined, PatentEye uses functionality provided by the CrystalEye webcrawler to interact with the EPO search interface. Where full-text XML is available for a relevant patent document, the corresponding ZIP file is retrieved. This is notably absent in the case of patents that have been published under the Patent Cooperation Treaty (PCT) instead of filed directly with the EPO.

In order to create a corpus for the current work, chemical patents from the EPO website for the ten weeks dated from 2009-05-06 to 2009-07-08 were downloaded. Duplicate patent documents were deleted such that only one document remained for each patent ID within the corpus, which then totalled 690 zipfiles. Of these 690 files, it was found that 23 did not contain the XML version of the patent under the expected file name. The subsequent work using the downloaded patent corpus is therefore based on a reduced corpus of 667 unique, full-text patent documents where the XML files are used as input.

### Enhancement of document semantics

**Figure 5:**
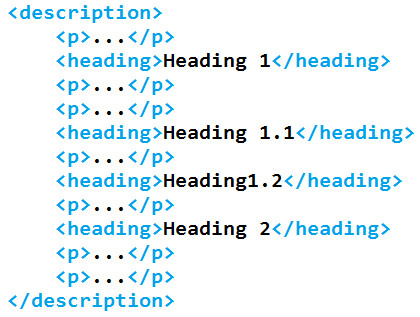
**Flat document structure as received from the EPO**.

As discussed previously, the different sections of the XML-formatted patent documents are not always clearly defined. The content of the description element is relatively flat-that is to say, the heading and p (paragraph) children are siblings of one another, such as in Figure 5.

To a human reader, it is a simple task to realise that the headings 1.1 and 1.2 are subsections of Heading 1, and that the each of the paragraphs belongs to a section of the document that begins with the preceding heading. Since this is not made explicit in the structure of the XML, however, it is not trivially obvious to a machine that the document should be read in such a way. For this reason it is desirable to deflatten the XML-to rewrite the document such that as much of the implicit structure is made explicit as possible. This rewritten document is then saved to disk in order to prevent unnecessary repetition of the task.

A number of other semantic enhancements are performed on the patent documents at this stage. These tasks include the application of OSCAR3 data recognition to identify spectral data within the text, the application of OSRA to add SMILES representations of the chemical structure images contained within the documents, the recognition and annotation of references in the text to other sections of the document, *e.g*. "the reaction was performed as in example 12" and the identification and labelling of the paragraphs in the text that form part of an experimental section.

#### Paragraph Deflattening

**Figure 6:**
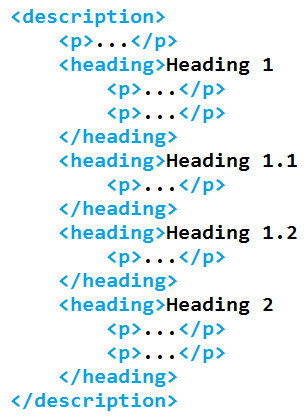
**Reordered document showing explicit structure**.

In this step, the description element of the patent document is checked for paragraph children. Any p elements that are found are detached from the document and re-attached as a child of the heading element that most recently precedes them. Any p elements that occur before the first heading child of the description element are ignored by this process. For example, the example of XML in the preceding section would be reformatted as shown in Figure 6.

**Figure 7:**
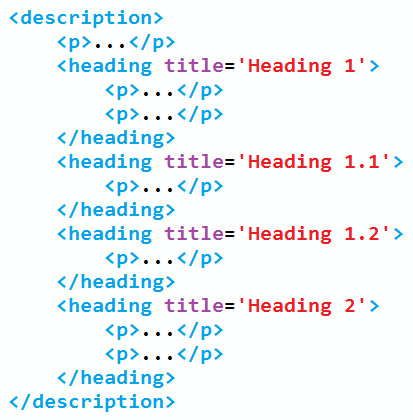
**Reordered document showing explicit structure and avoiding mixed content**.

Before this reformatting, the heading element was acting as an annotation on the heading text. While it can still be inferred that the text inside a heading element and preceding the first p element is the heading text, the reformatting process has destroyed the explicit declaration and created mixed content. To remove the requirement to infer the heading title, the heading text is removed from the document and made into a title attribute on the heading element, to form a document of the form shown in Figure 7.

#### Document Segmentation

**Figure 8:**
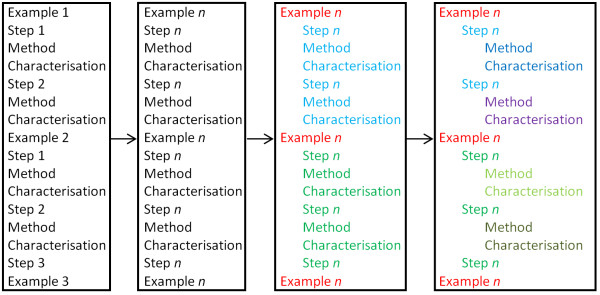
**Identification of and Document Restructuring Using Consecutive Headings**.

As previously discussed, the EPO do not attempt to explicitly demarcate in their XML the existence of sections of a patent document. Headings in the document are denoted by use of the heading tag, but otherwise the reader is left to infer for themselves where subheadings occur and to which headings they belong. This lack of formal structure in the document is a barrier to the automated processing of the patent documents as it prevents a machine from making context-specific decisions about how to behave. At this stage in the semantic enrichment process, an attempt is made to formalise the document's implicit structure. Firstly, those headings that correspond to primary document sections such as "technical field" or "description of embodiments" can be identified by matching using regular expressions based on those headings given in Figure 1 to permit slight variation on the author's part. These headings are renamed (*e.g*. to "disclosureOfInvention" or "summaryOfInvention") to enable trivial location of them within the document, and the child elements of description are reordered as previously so that headings that occur after a primary heading become a sub-heading of that primary heading.. Secondly, in an iterative process, lists of headings that form a consecutive list (*e.g*. "example 1" and "example 2") are identified by finding those headings that have identical text content, disregarding incrementable strings (*e.g*. "1" and "1a") and chemical names, as identified by OSCAR. The structure of the document is then rewritten to reflect the fact that a heading that intervenes in such a list is logical a subheading of the preceding heading. This process is illustrated in Figure 8.

#### Data Annotation

To facilitate its later use in the workflow, characterisation data is at this stage identified and annotated using the OSCAR3 data functionality. The text of each paragraph is passed to OSCAR3 for data recognition, which applies inline annotation to label the various parts of the spectrum. Where data is found, these annotations are inserted into the patent XML document in the appropriate places. In this way, the original text content of the patent document remains intact, and is rendered machine-understandable.

#### Classification of Synthesis Sections

While it is common for the experimental sections, *i.e*. those that describe the process and results of a chemical reaction, of a patent to occur as examples of the invention, it is not necessarily the case that the method of identifying document sections described previously will result in their occurrence as part of an example element in the semantically enhanced patent documents. As a result, the semantic enhancement at this point has done nothing to identify the presence or location of some or all of the experimental sections in a number of documents. To address this concern the sections of the text, as contained by opening and closing heading tags, are classified as being either *experimental *or *non-experimental *by use of a naïve Bayesian classifier. This classification is achieved using the third-party Java library Classifier4J [39], version 0.6., and allows for a greater proportion of the experimental sections within the patent corpus to be recognised as such and treated appropriately during the later stages of the workflow.

A corpus was assembled by selecting 800 p elements (*i.e*. paragraphs, in the most part) from those patents that had successfully passed through the paragraph deflattening and document segmentation phases of the semantic enrichment procedure, using a random process in which each paragraph had an equal chance of selection. These paragraphs were manually inspected and determined to be *experimental*, *non-experimental *or *empty *according to the following criteria;

• The paragraph is *empty *if it has no text content. Such empty paragraphs generally occur in the patent documents as containers for images.

• The paragraph is *experimental *if;

a. It is an account of a reaction or a part of a reaction, including by way of reference to another section of text *e.g*. "The reaction was carried out as in example 12".

b. It is a report of spectral or other characterisation data.

c. It is some combination of the above.

• The paragraph is *non-experimental *if it is not *empty *or *experimental*.

The manually-classified paragraphs may be summarised as follows (Table 2).

**Table 2:** Summary of manually-classified paragraphs

Class	Frequency of Occurrence
Empty	117
Experimental	238
Non-experimental	445

In order to produce experimental and non-experimental sets of equal size, non-experimental paragraphs after the 238^th ^occurrence were ignored for the remainder of this work. The first 119 (50% of the full set) experimental and non-experimental paragraphs were then used to train the Bayesian classifier before it was asked to predict probabilities of the remaining experimental and non-experimental paragraphs belonging to the experimental class. The predicted likelihoods may be summarised as follows (Table 3).

**Table 3:** Predicted probabilities of experimental and non-experimental paragraphs belonging to the experimental class.

Experimental		Non-experimental	
**Predicted likelihood**	**Frequency**	**Predicted likelihood**	**Frequency**

0.99	115	0.01	102
0.98 ≥ p > 0.95	1	0.01 < p ≤ 0.06	3
0.05 ≥ p > 0	4	0.06 < p < 0.5	2
		0.99	12

Thus, when classifying paragraphs as experimental if p < 0.5 and non-experimental if p > 0.5, the experimental paragraphs were correctly classified at a rate of 96.6% and the non-experimental paragraphs at a rate of 89.9%. These rates were deemed high enough to continue into production.

Heading elements in the patent documents are identified by use of the XPath "//heading". If a heading has text content, the text content is passed to the ParagraphClassifier for a prediction to be made. If the predicted likelihood is greater than 0.5, the section is classified as being experimental, and this is noted in the XML by the addition of a classifier4j attribute with the value "experimental". Otherwise, the opposite is recorded by setting the value of the classifier4j attribute to "nonExperimental".

#### Image Analysis

As previously discussed, the EPO patents frequently feature chemical structure diagrams that illustrate the example compounds for which syntheses are reported. These images therefore contain useful information that can be used to identify the product of a reaction. While USPTO patents supply the connection tables for such structures in the form of ChemDraw and MOL files, in the case of the EPO patents it is necessary to use image-to-structure software to interpret the supplied TIFF files. As the only such Openly available package at the time, OSRA was used for this task. As the most recent version available at the time that the work commenced, version 1.2.2 was employed. Applying OSRA to a chemical image resulted in a SMILES string, which was attached to the patent XML document as the value of an osraResult attribute applied to the img element in question.

In order to validate the performance of OSRA on the patent images, a corpus of two hundred images of single chemical structures was formed by random selection from the patent corpus. The chemical structures contained within these images were manually converted to SMILES strings, chiefly by redrawing the structure using ChemDraw 12.0 and exporting the structure as SMILES or by manual conversion in the case of simple structures, which were recorded in an index of the corpus. OSRA was used to analyse each of the 200 single chemical structure images, and the results of this analysis was appended to the index.

Previous authors in the field have suggested subjective metrics of success such as less than 30 seconds of human editing being required to correct errors in the structure [40], while Filippov and Nicklaus [28] propose measuring success by calculating a similarity metric between the machine-produced structure and the correct structure. Such measures are of limited utility in the present work; manual correction of structures or determination of correct structures cannot be implemented within a fully automated workflow. What is desired of the image analysis process is the correct identification of the product molecules of chemical syntheses, and while a high similarity between a structure believed to be the product (the "candidate product") and a structure produced by OSRA may be indicative that the image analysis has made a minor error and the candidate product should be accepted, it may equally indicate that the image analysis is correct and the candidate product should be rejected. As a result, there is no threshold of similarity below the two structures being identical at which the structure derived from the image analysis becomes "good enough".

The manually-generated and OSRA-generated SMILES strings for each image were thus used to generate the canonical identifier InChI using JUMBO [41]. The performance of OSRA was measured by comparing these InChIs by string equivalence; where the two InChIs were identical, it was counted as OSRA having correctly deduced the chemical structure contained within the image and considered a match. Where the InChIs differed it was considered a non-match. In a number of cases, it was not possible to generate an InChI from the SMILES string produced by OSRA. The causes of these problems were also examined and determined to be primarily that the SMILES string contained the wildcard character, *, which is valid SMILES but is not supported by the JUMBO SMILES parser. In a further two cases the SMILES string returned by OSRA was found not to be valid, suggesting a bug within the OSRA program itself.

The results from this work were as follows (Table 4).

**Table 4:** OSRA performance

Result	Frequency	%
Match	68	34.0
Non-match	79	39.5
Unbuildable SMILES (containing wildcard)	51	25.5
Invalid SMILES	2	1.0

**Figure 9:**
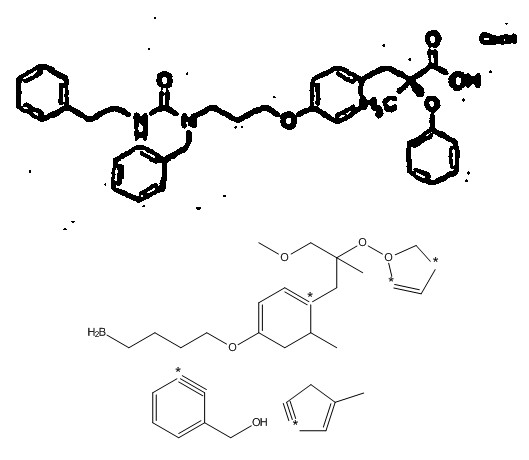
**Input image (top) and unbuildable result (bottom)**.

The agreement between the OSRA-produced structure and the manually-produced structure is, at 34%, significantly lower than that reported for OSRA 1.1.0 by Filippov and Nicklaus [42], in which the rate was reported as 26 matches out of 42 (61.9%) structures and 107 matches out of 215 (50.0%) structures on two data sets. Such rates will of course be highly dependent upon the images that form the test corpus, and the images supplied by the EPO are of highly variable quality. Many of the images that form the test corpus used in this work are severely pixelated, indistinct or contain background noise; some are only barely legible to a human skilled in the art. Such an example, together with the structure as interpreted by OSRA, is shown in Figure 9.

### Extraction of reactions

Chemical patents are a rich source of chemical reactions due to the requirement for a patent claimant to detail examples of the invention. The reactions published in this way are routinely manually indexed and added to databases such as CASREACT. In order to devise a system for automated extraction from reported syntheses, it is important to first consider the nature and common structure of such text. Fortunately, the reporting of chemical syntheses is highly stylised. By convention, chemists report syntheses using the past tense and the agentless passive voice, which simplifies the process. Descriptions of syntheses may be conceptually divided into three parts-the *primary reaction*, in which the target compound is completely or substantially produced; the *work-up*, in which the reaction is quenched and neutralised, solvents are removed, the product purified and suchlike; and the *characterisation*, in which spectral data is afforded to demonstrate that the product of the reaction is that intended. In the description of the primary reaction, reactants ("a substance that is consumed during the course of a reaction" [43]) are detailed by giving a name or other reference (*e.g*. "ketone 12b" or "the compound from step 2") together with the quantity used, generally stated by mass and by molar amount. Solvents are typically detailed by giving a name and the volume used. In the description of the work-up these quantities are commonly omitted. The identity of the product of the synthesis may be specified in one of two typical ways; in the heading of the section, or by statement at the end of the description of the work-up, *e.g*. "to yield 1,6-naphthyridine-8-carboxylic acid".

**Figure 10:**
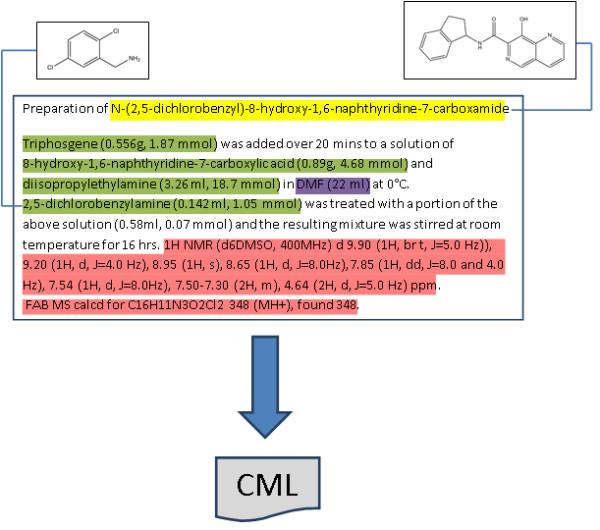
**Schematic workflow diagram illustrating the extraction of reactions from the patent content**. Text is analysed to identify the title compound (yellow), reagents (green), solvents (purple) and data (red), chemical names are used to construct connection tables and the reaction is saved as CML.

The enhanced patent XML documents are read into memory, and the headings that have been classed as experimental by the ParagraphClassifier or that are descended from example headings are identified by means of XPath. The sections of the document either contained within the heading or example element or, if the heading has sub-headings, each subheading individually, are passed into the ExperimentParser class. Identities and amounts of reagents are identified by analysis of the text using ChemicalTagger. Spectral data in the text have been previously annotated using OSCAR3, and NMR spectra present are converted to CML using a converter from JUMBO-Converters [44]. The product of the reaction is identified by using OSCAR3 to identify chemical names in the heading title. The product identity is then validated by comparison with the results of the OSRA analysis of any image present, and with any ^1^H NMR or mass spectrum that is reported. The results of these processes are combined into a CML Reaction which is saved to disk. This workflow is expanded in greater detail in the following sections and summarised graphically in Figure 10.

#### Identification of reagents

Reagents used during the primary reaction section of a chemical synthesis are, by convention, reported along with the quantity used. Such lexical patterns are easily identified using ChemicalTagger.

#### Identification of products

In order to identify the product of a reaction, the title text of the document section under examination is passed to OSCAR3 for named entity annotation. If OSCAR3 does not identify a single chemical name (CM) in the title text, then the process of reaction extraction fails and the ExperimentParser throws a RuntimeException. If a single CM is found in the title text, then the name is resolved to a CML Molecule, which is added to the productList of the CML Reaction.

#### Attachment of spectral data

The most common spectra types found in the patent corpus were ^1^H NMR, ^13^C NMR and mass spectra. The reports of mass spectra generally report only the mass of the molecular ion, optionally plus or minus a defined offset, and so provide a useful source of information for validating a candidate product molecule but little information worth preserving. The NMR spectra, however, in addition to providing a means by which the product molecule may be verified, are themselves data of potential importance and are worth preserving for future re-use. The format in which they are preserved in the enhanced patent XML documents, using inline annotation to identify features within the original patent text, is ideal in that context as it retains the original document text. It does not, however, enable trivial machine interpretation of the spectrum since it is not valid CML and tools do not exist for its easy manipulation. The OSCAR-annotated spectrum is therefore converted into a CML Spectrum by use of the OSCAR2CMLSpectConverter class in the JUMBO-Converters library. It does not attempt to perform any further text-mining on its input, instead relying entirely on the OSCAR3 annotations to fully identify features of interest such as peaks, integrals, multiplicities and coupling constants.

#### Automated verification of product-checking against embedded images/mass spectrum/^1^H-NMR

It is desirable to be able to automatically verify the product in some way. This can be achieved by comparing the determined product to the extracted spectral data and, if present, any accompanying chemical images. The process of acquiring these sources of information must also be regarded as potentially inaccurate, and so it is not possible to definitively confirm or refute any candidate product. Nonetheless, these checks provide potentially useful information regarding the validity of the assigned product and of the assigned spectral data.

Given the ^1^H NMR spectrum of an unknown compound, it is possible for one skilled in the art to discount certain candidate structures. Most trivially, the proton count in the candidate structure should agree with total integral of the NMR spectrum. Each unique chemical environment in the candidate structure should give rise to a distinct peak in the NMR spectrum and it should be possible to assign for each of the chemical environments a peak that is in the correct region of the NMR spectrum. The peak multiplicities should be explained by potential couplings in the candidate molecule, and protons that couple to one another should share coupling constants. The application of these rules is subject to a large amount of subtlety, however. While each chemical environment should give rise to an individual peak, these peaks can overlap and be indistinguishable from one another, most notably in the case of aromatic protons. The determination of unique chemical environments is complicated by the need to consider three dimensional effects, such as in the case of two protons sited on inequivalent faces of a ring system. As a result of these effects, it is not possible to compute chemical environments based solely on the 2D connectivity of a molecule and confidently assert that this will equal the number of peaks reported in the molecule's NMR spectrum. Whether there are more or fewer peaks in the NMR spectrum than predicted, the candidate molecule may be correct. Conversely, if the prediction matches the observation the candidate molecule may still be incorrect. The resolution of this problem falls outside of the scope of this project, and so the checking of structures against ^1^H NMR spectra is limited to the first method mentioned-ensuring that that the proton count of the molecule agrees with the total integral of the spectrum. The result of this check is recorded in the automatically generated CML Reaction by adding a matchesHnmr attribute to the product molecule, with a value of true or false as appropriate. Where a mass spectrum has been recognised, isotopomer masses for the candidate product structure are calculated using JUMBO and compared to the reported mass and the results of this check are recorded as before, using the matchesMassSpec attribute.

When the experimental section includes a chemical image it is possible to compare the connection table of the candidate product with the results from the OSRA analysis of that image. If a chemical image is found within the source experimental section, the recorded SMILES strings for the image are built into CML Molecules which are then used to generate InChIs, using the SMILESTool and InChIGeneratorTool classes from JUMBO respectively. An InChI for the candidate product molecule is similarly generated, and the InChIs are subsequently compared.

Since the image included in the experimental section may be a reaction diagram it is possible for OSRA to have identified more than one molecule. Since the analysis of the often low-quality images is an error prone procedure, it is possible that the structures identified in the image may not contain the correct product. As previously discussed, when OSRA fails to correctly deduce a connection table from a drawn structure, it frequently reports a result containing the wildcard character, "*". This character is not recognised by JUMBO's SMILESTool, causing it to throw an Exception. As a result, the following rules are applied when checking the candidate product against embedded images:

1. If the InChI generated for the candidate product matches one generated for the structures identified by OSRA, the product is considered to match the image.

2. If all of the structures identified by OSRA can be built into InChIs and the InChI for the candidate product does not match one of these, the product is considered not to match the image.

3. If some or all of the structures identified by OSRA cannot be built into InChIs and the InChI for the candidate product is not matched by one of those generated from the chemical image, no conclusion is drawn.

If a conclusion is drawn from this process, it is recorded in the CML Reaction by the addition of a matchesImage attribute on the product CML Molecule, with a value of true or false as appropriate. If no conclusion is drawn, or if the source experimental section does not contain a chemical image, the CML Reaction is not modified.

### Performance of the Reaction Extraction Process

Using the methods described above, 26287 input sections for the reaction extraction procedure were derived from the corpus of 667 patent documents. From these inputs, a total of 4444 CML Reactions were derived, representing around 17% of the total input. The principle causes of failure to generate a CML Reaction included the input section containing no text-containing paragraph children; the input section containing more than one text-containing paragraph children (in which case the system backs off since this may describe a single or a multi-step reaction); the failure to identify the product molecule; and the failure to identify any reagent phrases in the source text.

To assess the accuracy of the semantified reactions, the output of the reaction extraction process was manually examined and compared with the source text. Each CML Reaction was assessed on a number of criteria to determine the performance of the different modules of the reaction extractions system. These criteria included the accuracy of identified products, reagents and spectra, and the performance of the systems for automated product verification was tested by comparing the results of the automated verification with those of the manual verification. The methods employed for this process and the results obtained are subsequently discussed.

Since the manual inspection of each and every reaction extracted from the patent texts was not a feasible task, a subset was selected to serve as a corpus from which to derive performance metrics. From the 4444 reactions successfully extracted from the 667 unique, full-text patent documents, 100 reactions were selected at random. This reaction corpus was then used in the subsequently described validation procedures.

During the manual inspection of the reaction corpus, it was discovered that two of the 100 CML Reactions were derived from multi-step syntheses that were described within a single paragraph. Since these cases did not reflect the kind of input for which the current software was designed, they were excluded from the analysis process. A further two CML Reactions in the reaction corpus were found to have been derived from examples of their respective inventions that did not describe chemical syntheses-instead describing assays. These CML Reactions were similarly excluded from the analysis process; consequently, the process is based upon a reduced corpus of 96 CML Reactions.

The source from which the reaction was extracted was examined to determine whether the chemical name identified in the heading text by OSCAR3 and from which the product CML molecule was generated agreed with that stated in the heading text. Since the name to structure conversion process is not a perfect procedure, this is no guarantee that the attached connection table is also correct. However, the development of OPSIN was not a part of the current work and is reported to operate at an extremely high rate of performance [22] and so it was not considered necessary to measure the accuracy of this process. The manual inspection of the reaction corpus showed that the correct product was identified on 88 of the 96 occasions, a success rate of around 92%. It was further noted that on each of the 8 occasions on which the correct product was not identified, the term identified as the product name could not be successfully resolved to a connection table, suggesting a means by which the errors may be automatically removed. Generally, the cause of the failure to identify the correct product was due to the product of the reaction being named in the accompanying text, and hence not being present in the section heading of the source; instead, a term from the heading was falsely identified as a chemical name, which allowed for the creation of a CML Reaction from the source.

The sources from which the reaction corpus was extracted were examined, and for each the reagents employed and the amounts thereof were identified. These were then compared with those automatically extracted; instances where the same chemical name and amount were both manually identified and automatically extracted were counted as true positives, where the automatically extracted reagent list contained an instance that was not matched by both chemical name and amount in those manually identified a false positive was counted, and where a reagent was manually identified that was not automatically extracted, a false negative was counted.

This work required the formalisation of the concept of a reagent to a sufficient degree that any subjectivity in determining what did and did not constitute a reagent could as far as possible be minimised. The IUPAC definition, "a test substance that is added to a system in order to bring about a reaction or to see whether a reaction occurs" [38], does not match the common usage of the term which further includes the chemical species involved in a reaction, *i.e*. reactants, solvents, catalysts, *etc*. It is this wider definition that fits the goal of the current work-to automatically determine how a reaction is carried out.

It was observed when considering this task that the chemical literature frequently underspecifies the work-up stage of a reaction. That is to say, the reagents employed may be stated without reference to their amounts, such as in;

*"The reaction mixture was stirred at 25°C for 4 days and then diluted with ethyl acetate. The mixture was then washed with a dilute aqueous hydrochloric acid solution. At this time, methanol was added to the organic layer. A precipitate formed and was removed by filtration. The organics were further washed with a saturated aqueous sodium chloride solution, dried over magnesium sulfate, filtered, and concentrated in vacuo. The resulting solid was triturated with diethyl ether. The solid was collected by filtration and washed again with diethyl ether to afford..." *[34]

While the work-up is an undeniably important phase of a reaction, the techniques used in the current work are reliant on the specification of amounts in order to identify reagents. This technique is well-suited to identification of primary reagents but not those used in work-up, and so in order to produce a metric that indicates the performance of the software in the role for which it was designed it was decided to entirely omit reagents mentioned in the work-up phase, and inert atmospheres under which reactions were performed, from the current analysis.

The manual inspection of the reaction corpus identified 249 true positives, 71 false positives and 139 false negatives-the system having a precision of around 78% and recall of around 64%. When considering these results, it should be remembered that the requirement for an identified reagent to be considered a true positive-that not only the chemical name but also the amounts employed in the reaction be identical to those described in the source text-is a rigorous standard. It was commonly the case during the analysis that the system identified the correct chemical name as a reagent but failed to correctly add one or more amounts, creating both a false positive and a false negative. These situations occurred where one or more of the amounts in the source text were not recognised by ChemicalTagger. Frequently these situations were caused by the patent author employing a structure that may be considered incorrect, *e.g*. "triphenylphosphine (3.08 g., 11.78 mmol)" or "1-Phenylpiperazine (16.2 g, 0.10 mole)". The non-standard full stop indicating the abbreviation of "grams" in the first example and the failure to contract the unit "mole" to its standard symbol "mol" in the second result in the failure to recognise and convert these amounts to CML. The data gathered in the current exercise permit the improvement of the ChemicalTagger grammar to recognise a greater variety of the reporting formats used by authors and thereby improve the precision and recall for the identification of reagents as measured by the current methods.

These improvements, however, are not sufficient on their own to produce a system that operates at the level of a human operator. The current system requires further development before the data it produces are of sufficient quality to be considered reliable by the community at large.

The extracted reactions contain, where identified and successfully converted to CML, the ^13^C and ^1^H NMR spectra of the products. In the patents used for this work, ^1^H NMR spectra are far more common than ^13^C-indeed, the manually examined subset of the reaction corpus was found to contain only two ^13^C NMR spectra. Consequently, only the validity of the attached ^1^H NMR spectra in the reaction corpus was considered. Where these spectra were present, the content was compared to the reported spectra in the original sources. In order to be considered correct, the attached spectra were required to fully describe the original spectra in terms of the shifts, integrals, multiplicities and coupling constants of each peak-any deviation from what was reported in the original text resulted in the attached spectrum being judged to be incorrect.

The manual inspection identified 25 occasions on which the ^1^H NMR spectrum attached to a product molecule precisely replicated the information presented in the source text and 8 occasions on which it did not, *i.e*. a success rate of around 76%. The primary causes of the inclusion of incorrect ^1^H NMR spectra were the failure to fully convert peak metadata, *e.g*. multiplicities, as identified by OSCAR3 to CML and the conversion to CML spectra of sections of input text that did not indicate ^1^H NMR spectra, *i.e*. false positives in the data recognition procedure. The first of these issues indicates a bug in JUMBO-Converters that could be relatively trivially identified and fixed while it is expected that the second issue should produce ^1^H NMR that could be automatically distinguished from a genuine NMR spectrum in a majority of cases, since false positives will rarely contain expected peak metadata such as integrals and multiplicities. Though the ^1^H NMR spectra validation is based on a small set of data, it is believed that the spectra identified by PatentEye are of nearly sufficient quality that they constitute a resource of value to the community.

## The Green Chain Reaction: are chemical reactions in the literature getting greener?

The development of an automatable patent extraction and interpretation system gave us an opportunity to include the scientific community in an *ad hoc *public project. ScienceOnline (2010) was a gathering of bloggers, information specialists, information providers, publishers and funders related to the communication of science. We set up a one month project dedicated to providing "a scientific result" by the time of the meeting. This highly ambitious idea relied on a critical mass of collaborators in a virtual community installing programs, running them to collect chemical information and aggregating it for presentation at the meeting.

The focus of the experiment was to see if a well-defined question could be answered by extracting information from the patent literature, using PatentEye.

As the basis of the project, named "The Green Chain Reaction" (GCR), we chose to focus on the use of solvents in chemical reactions to determining the "greenness" of chemical reactions in manufacturing and research. Traditional methods of chemical synthesis are becoming increasingly unacceptable because the processes are hazardous (explosion, toxicity), they consume scarce resources (metals, petrochemicals, *etc*.), the by-products (unwanted materials, which are often discharged to the environment) are hazardous (toxic, *etc*.) and they are energy-intensive. Both machines and humans were employed to collect and systematize chemical syntheses and to analyse the results.

The Green Chain Reaction was "Open Notebook Science" in that all discussions, code and results were publically viewable on the web at all stages. Moreover, anyone could volunteer to participate in the project. The planned methodology was:

1. A volunteer downloads the GCR software and installs it on their machine.

2. They run it against a given week of patent data from the 500 weeks available on the EPO website.

3. The software analyses the occurrence of solvents in the patents and records each instance of a particular solvent.

4. The software provides an aggregation and uploads this to a common site (an Open server at Cambridge).

5. The Cambridge software makes a further aggregation and presents the results.

In one sense this is a human analogy of a map-reduced project where a given task is farmed out to a large number of "computers" and the results are aggregated. In practice, we found a number of problems in distributing the software. The OSCAR package did not run "out-of-the-box" on all architectures, and it was some time before we discovered the cause of this (OSCAR's workspace). For this reason some volunteers were not able to participate in the complete project. As we discovered bugs, new releases were made, sometimes on a daily basis. Nevertheless, we were ultimately able to analyse about 100,000 patents and to tabulate the results.

The GCR PatentEye workflow is as follows:

• Analyses a weekly patent index and downloads all the chemical patents

• Trawls through the patents to see which contain experimental sections

• Analyses the text to extract mentions of solvents, including chemical formula and amount (where given)

• Aggregates all the solvent data from a single patent into a summary file (dissolveTotal.html)

• Uploads the summary file to the GreenChainReaction website http://greenchain.ch.cam.ac.uk/patents/results/

The results were communicated onto the Cambridge server using a RESTful process. The solvents were identified by their linguistic context (using ChemicalTagger), and validated against Wikipedia pages of the same name. Thus, for example, ethyl acetate would have been determined as a solvent because of its linguistic environment (*e.g*. "dissolved in ethyl acetate" or "in 50 ml of EtOAc"). Sometimes the solvents were given as textual names (*e.g*. dichloromethane), and sometimes as compositional formulae (*e.g*. CH_2_Cl_2_). The first observation is that the extraction of solvents is extremely high precision *i.e*. there are very few entities retrieved which are not solvents. We have no information about the recall but it is clear that a large amount of data has been extracted. The solvents were then listed on the server with their aggregate counts and the chemical structure diagram retrieved from Wikipedia. Note that there needs to be a further disambiguation of names, so that there are entries for both dichloromethane and CH_2_Cl_2_, which should be summed, but in the time available for the project and with the given volunteers it was not possible to include this stage. The precision would appear to be > 99.9%.

We had hoped that there might be a large change in solvent usage over a decade. However, the most commonly used solvents (THF, dichloromethane) have remained at approximately the same frequency. These solvents are not completely green being a) potentially explosive and b) containing toxic C-Cl bonds, so there is no particular evidence in increasing greenness. However we caution this interpretation as there are many dates associated with the patent, and we cannot be sure how these relate to the actual dates on which the syntheses were carried out. Moreover there is a considerable lag between the actual synthesis and the publication of the patent so that recent changes in use have probably not been picked up.

## Competing interests

The authors declare that they have no competing interests.

## Authors' contributions

DMJ wrote the manuscript, developed the PatentEye software and carried out the analysis of its performance.

SEA provided structure analysis routines, helped develop the Green Chain Reaction software, advised on software architecture and helped write the manuscript.

PMR developed OSCAR and CML, organised and ran the Green Chain Reaction experiment and wrote the manuscript.
